# Locating the potential development of spatial ability in the Swedish national curriculum

**DOI:** 10.1016/j.heliyon.2024.e38356

**Published:** 2024-09-24

**Authors:** Ting Jun Lin, Jeffrey Buckley, Lena Gumaelius, Ernest Ampadu

**Affiliations:** a Department of learning, KTH Royal Institute of Technology, Stockholm, Sweden; b Technological University of the Shannon, Midlands Midwest, Athlone Campus, Westmeath, Ireland

**Keywords:** Spatial ability, national curriculum, Swedish compulsory education, Content analysis

## Abstract

There exists a plethora of studies that have related pupils’ spatial ability to their academic achievement and problem-solving skills, especially in science, technology, engineering, and mathematics (STEM) subjects. However, little is known about how spatial ability could be presented in a national curriculum. To increase the awareness and intention to develop pupils' spatial ability within the national curriculum, the compulsory curriculum document from the Swedish National Agency for Education which details all subject syllabi is examined and analysed using a content analysis method. The results show that two major dimensions could be used to locate the potential spatial ability development within the curriculum. The first is a visual dimension, which manifests as three different visual components: graphical, pictorial, and manufactured. The second is an epistemic dimension, which describes how pupils' different types of spatial knowledge can be nurtured, and inductively described as conceptual, procedural, and spatial citizenship knowledge. A three-by-three matrix framework is created based on the above dimensions and components. Policymakers and educators in Sweden, as well as in other countries, may draw insights from the framework created by this study and adapt it to educational practice, particularly in the realm of developing students' spatial ability through curriculum design and classroom instruction.

## 1 Introduction

We immerse ourselves in a spatial world where we interact with two- and three-dimensional objects [1]. The ability that enables us to engage with the three-dimensional world is referred to as spatial ability. Spatial ability permeates our daily-life problem-solving process such as when reading maps, navigating directions, or simply assembling and rearranging furniture in a room. As a critical factor of human intelligence [[2], [3], [4]], spatial ability has been linked to educational success generally [2,5]. Most notably, it predicts engagement and success in the fields of science, technology, engineering, and mathematics (STEM) (e.g. Refs. [6,7]). These subjects often require spatial ability. For example, perceiving and counting the sides of a two-dimensional object in mathematics learning requires the abilities of visualization and object combination [8]. In science learning, the ability of visual-motor coordination is required when students need to conduct hands-on activities [9]. Educational researchers have found that spatial ability is malleable (e.g. Refs. [10,11]). With appropriate interventions for spatial ability, educators can improve students’ performance in many STEM subjects [7,10]. Hence, spatial ability has been integrated into many school curricula for different age groups. In some countries, educational researchers have been making efforts to scale up spatial ability development to a national level such as the United States (US) (e.g. Ref. [12]), the United Kingdom (UK) (e.g., Ref. [13]), and Ireland [14,15], where many students in the compulsory school system are currently undergoing specialized curricula which focuses on developing spatial ability.

In Sweden, spatial ability is implied in the national curriculum document that is issued by the Swedish National Agency for Education [16]. Through the lens of geography, the discussion from Örbring [17] was one of the first to comment on spatial ability development in conjunction with the Swedish national curriculum. According to the study [17], spatial ability is implicitly expressed in the Swedish national curriculum. It is implicitly required as a tool to acquire some geographical capacities such as interpreting and visualizing locations. It is assumed that geography teachers should be capable of translating the implicit expression in the curriculum document into spatial ability development and utilization in teaching. Hence, by concluding the study, Örbring [17] prompted stakeholders to ponder if more explicit depictions of spatial ability in the Swedish national curriculum would promote geography teaching in Sweden. In a similar vein, another study by Lin et al. [18] established a methodological framework to examine how spatial ability is situated in the Swedish national curriculum, specifically, in the syllabi of Craft and Technology. Their framework highlights, for educators and researchers, the potential of interpreting the Swedish national curriculum to guide teachers' enacted practice towards the development of pupils’ spatial ability along with teaching the subjects of craft and technology.

The above studies motivate the current study to further investigate the representation of spatial ability in the Swedish national curriculum. Understanding where spatial ability could be fostered through the national curriculum can provide a guide for the augmentation of pedagogical practice such that, in addition to receiving the national curriculum, spatial ability can also be purposefully developed. In this study, we refer to the curriculum as the written formal curriculum that is issued by the official agency for education in the form of guidelines, syllabi, and associated materials that direct and support compulsory education in the country. We build upon the above studies and extend the examination to all of the syllabi in the current version of the Swedish national curriculum [16], with the aim of addressing the research question: how can spatial ability be potentially identified and interpreted in the Swedish national curriculum? From this study, it is envisioned that, through increased awareness, the development of spatial ability within the national curriculum could be promoted in Sweden.

## 2 Literature review

### 2.1 The conceptualization of spatial ability

In this study, we adopt the Cattell-Horn-Carroll (CHC) theory as the conceptualization of spatial ability as “the ability to make use of simulated mental imagery to solve problems—perceiving, discriminating, manipulating, and recalling nonlinguistic images in the ‘mind's eye’” ([19], p.125). The CHC theory has held an enduring legacy of nearly three decades [3], and it is one of the most impactful theories regarding human intelligence. Researchers have identified various dimensions of spatial ability and developed frameworks regarding how it manifests in practice (e.g. Refs. [5,20,21]). To date back, McGee [22] suggested that spatial ability should include spatial visualization and spatial orientation, where spatial visualization entails manipulating objects in the mind, and spatial orientation involves perceiving objects from various perspectives. Lohman [23] later added the dimension of speeded rotation, which involves promptly identifying the rotated versions of objects. Carroll [24] further concluded five main dimensions of spatial ability, namely, visualization, spatial relations, closure speed, closure flexibility, and perceptual speed. Building upon these dimensions, several contemporary frameworks have been developed and adopted today in applied educational research. These prominent frameworks include Newcombe and Shipley's [25] typology that bases spatial ability on the intrinsic-extrinsic and static-dynamic dimensions, Buckley and colleagues' [5] heuristic framework that describes 25 spatial factors to be translated into educational practice, and Posamentier and colleagues' [26] spatial model that offers eight elements for educators to “diagnose, train, and foster one's visual perception and spatial abilities” (p.25).

### 2.2 Spatial ability and academic achievement

Spatial ability has been repeatedly found to be a malleable and predictive variable of academic achievement across many previous studies (e.g. Refs. [7,10,27]). Some of the most prominent evidence comes from Wai and colleagues' [28] study. They tracked 400,000 high school students for over 11 years and found that students who excelled in spatial ability were more likely to pursue higher education credentials and professions in the STEM fields. The findings also suggest that spatial ability could discern potential talents for STEM students who might have been missed in contemporary talent identification [28,29]. In a more recent longitudinal study with 3948 first-year college students, Sorby and colleagues [7] revealed that explicit spatial ability training had a strong and positive effect on college students' engineering problem solving and analysis skills. Their intervention led to a significantly higher retention rate in engineering for female students. The study suggested that this could be because female students' enhanced spatial ability further unlocked their mathematical potential, which played an important role in their engineering learning [7]. For younger students, Judd and Klingberg [11] investigated the effect of spatial ability on mathematical learning among 17,648 children aged 6–8 years old. They found that children's mathematical learning was significantly increased after a 7-week training intervention on spatial ability. Specifically, spatial ability training on non-verbal reasoning and visuo-spatial working memory exerted the strongest positive effect on children's mathematical learning.

STEM subjects often require spatial ability, such as mathematics (e.g. Ref. [30]), geology (e.g. Ref. [31]), physics (e.g. Ref. [32]), and engineering (e.g. Ref. [33]). Researchers have been exploring how to better support students’ 10.13039/501100004527STEM learning in the 21st century by developing their spatial ability (e.g. Refs. [34,35]). Moreover, despite the smaller number of relevant studies, spatial ability has also been found to play an important role in non-STEM subjects. For example, in language learning, students might practice visualization and form constancy when they identify letters or words on printed paper such as posters, and recognize letters or words in various fonts among other pictorial features [26]. In music learning, especially in instrumental practice, the spatial nature of the music notation could expose students to pattern recognition and position matching between the notation and the instruments [36]. Therefore, it is pertinent for this study to examine both STEM and non-STEM subjects in the curriculum, providing holistic and meaningful insights for diverse stakeholders.

### 2.3 Spatial ability in the national curriculum

Spatial ability is garnering national attention in many countries since its critical role in students' academic achievement was recognized. Early in 2005, the 10.13039/100013101National Research Council (NRC) of the United States (US) released a report titled *Learning to Think Spatially—**GIS* *as* Support *System in the K-12 curriculum* [12]. The report initiated the need to develop K-12 students' spatial ability across the curriculum and to train teachers to teach and assess spatial ability in the country [12]. In more recent years, the National Science Foundation (NSF) of the US has been seeking opportunities to integrate spatial ability into the curriculum for a broader educational context. For example, the *ThinkSpace* curricular project was founded to support middle school students’ spatial ability development so that they can perform better in the astronomy curriculum [[37], [38], [39]]. Other projects regarding spatial ability within the curriculum were conducted in a higher education context. For example, three universities in the US have initiated a joint effort to integrate spatial ability training into the first-year college computing curriculum, to enhance the computing performance as well as the retention in the computing fields for female and underrepresented minority student populations [40].

In the UK, Bates and colleagues [13] have been developing a toolkit for educators to add spatial ability to the mathematics curriculum for young children. In Ireland, as the first phase of a national spatial ability project, researchers have collected baseline data from first-year secondary students across the country in terms of their spatial ability. The project partly aims to modify the national curriculum by integrating with spatial ability training programmes into it [14,15,41]. In an even broader context, a joint European project—Spatially Enhanced Learning Linked to 10.13039/501100004527STEM (SellSTEM)—based in 8 European countries was set up to support 10.13039/501100004527STEM learning through the role of spatial ability. The SellSTEM project has been working on uncovering the enablers and barriers to developing spatial ability, providing strategies for improving policies and curriculum, as well as offering teacher training for better spatial ability teaching [42,43].

Relevant initiatives seem to be scarce in Sweden. Interestingly, in the national curriculum document issued by the Swedish National Agency for Education (Skolverket), the term “spatial” is explicitly written only in the syllabi of Art, Sign Language for the Hearing, and social studies subjects such as Geography, History, Religion, and Civics. For example, the social studies syllabi for pupils of years 1–3 require teaching to cover the following content “*Spatial conditions in nature and the environment for population and settlement, such as land, water and climate*” ([16], p.221). The statement echoes what previous researchers termed “spatial citizenship”, which refers to a type of citizenship where an individual can interpret spatial information to deliver location-specific opinions and participate in society [44].

### 2.4 The two-dimensional framework

Lin and colleagues [18] suggested two dimensions through which researchers could locate potential spatial ability development within the Craft and Technology syllabi in the Swedish national curriculum. The first dimension is termed the visual dimension, which contains graphical, pictorial, and manufactured components [18]. The second dimension refers to how students construct their spatial knowledge, which includes conceptual and procedural knowledge [18] (Table 3 in the Methodology section of this study explains the definitions of each component of the framework). To locate the potential for spatial ability development in the curriculum document, Lin and colleagues [18] suggested that teachers could first identify a visual component in the syllabus such as “handicraft” (i.e., manufactured component) or “computer structure” (i.e., graphical component), and then understand what type of knowledge the curriculum intends to develop. As suggested by the study [18], teachers should engage pupils more in procedural knowledge, which might be more effective in increasing pupils’ spatial capacity and further leading to better performance in Craft and Technology. Indeed, procedural knowledge manifests through visual-spatial integration skills, which refers to the ability to conduct a series of cognitive process: (a) understanding and interpreting visual information from the surroundings, (b) integrating the visual information by building up a visual representation in the mind, and (c) representing the visual representation through performing muscle movement [45].

This study will extend Lin and colleagues’ [18] two-dimensional framework to present teachers of all subjects with an interpretation of where there is potential for spatial ability development across the Swedish national curriculum. Policymakers and educators in Sweden, as well as in other countries, may draw insights from the framework created by this study and adapt it to educational practice, particularly in the realm of developing students' spatial ability through curriculum design and classroom instruction.

## 3 Methodology

### 3.1 The Swedish curriculum document

We examined the Swedish national curriculum—*Curriculum for the Compulsory School, Preschool Class and School-age Educare* [16]. The document includes five chapters, which are *Fundamental values and mission of the school, Overall objectives and guidelines, Preschool class, School-age educare,* and *Syllabuses*. As the aim of this study is to situate potential spatial ability development in the taught subjects that pupils learn (pupils ages ≈ 7–16 years old), only Chapter 5 *Syllabuses* was included in the analysis. The chapter describes the subject aim, core content, and knowledge requirement for pupils at three grade stages (i.e., years 1–3, years 4–6, and years 7–9).

### 3.2 Content analysis

**Fig. 1:**
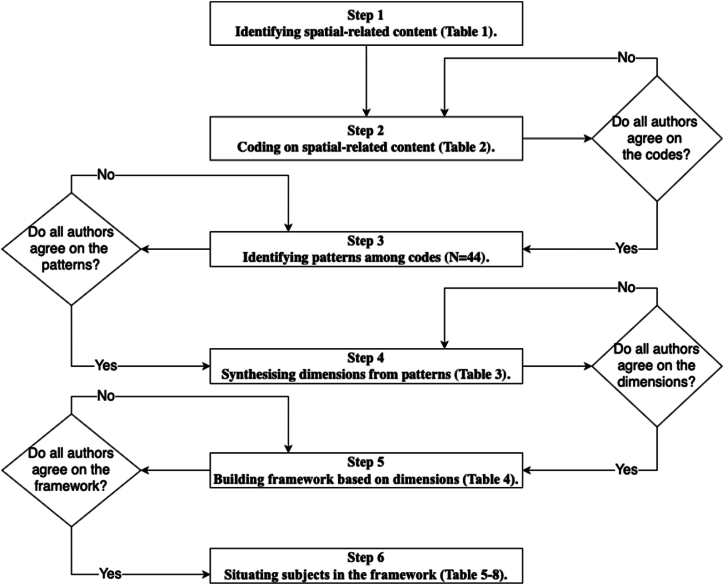
Flow chart of the methodological procedures.

Content analysis with both qualitative and quantitative methods was employed, and this process was supported by the analysis software NVivo. Qualitative coding was conducted to locate the relevant content that could be potentially interpreted into spatial ability development, followed by a quantitative frequency analysis to illustrate the occurrence of the potential spatial ability development across subjects. An overview of the methodology is presented in Fig. 1, and the following sections describe each step in detail.

The authors of this study possess extensive expertise in the fields of spatial ability research as well as curriculum studies: two of the authors are university professors in Sweden with knowledge of curriculum development, lending critical insights into the Swedish national curriculum to this study; one of the authors is a university lecturer, holding a PhD degree in the topic of spatial ability and STEM learning, bringing experience in conducting spatial ability research; lastly, another author is a PhD student, also specializing in the topic of spatial ability and STEM learning, who contributes to this study with much research training on the topic. For the procedures (Fig. 1), two authors undertook the process of identifying and coding the spatial-related content, followed by collective review and revision of the codes by all authors together. Constant meetings among all authors were held to address disagreements regarding each procedure until the final framework was established.

First, we read and examined the relevant sections of the document line by line, and we identified the content that could be potentially interpreted into spatial ability development. The identified content hereafter refers to the “spatial-related content”. Table 1 shows examples of identifying the spatial-related content from the curriculum document.

**Table 1:** *Examples of identifying spatial-related content*.

Curriculum content	Spatial-related content
**Outdoor life and other outdoor activities** • ^a^ Orientation in the local environment and the structure of simple maps.^b^ Concepts that describe location, distance and direction. • Exploration of possibilities for and realisation of games, physical activities and spending time in nature and other outdoor environments. • The basics of the right of public access. (Skolverket, 2024, p.182).	^a^ Orientation in the local environment and the structure of simple maps. ^b^ Concepts that describe location, distance and direction.
**Chemistry in nature, society and the human body** • ^c^ The structure, cycle and indestructibility of matter visualised using particle models.^d^ Elements, molecular and ionic compounds and how substances are transformed through chemical reactions.^e^ Atoms, electrons and nuclear particles. (Skolverket, 2024, p.169).	^c^ The structure, cycle and indestructibility of matter visualised using particle models. ^d^ Elements, molecular and ionic compounds and how substances are transformed through chemical reactions. ^e^ Atoms, electrons and nuclear particles.

Second, we coded the spatial-related content following Lin and colleagues’ [18] coding methodology. Specifically, both in-vivo [46] and descriptive coding methods [47] were used in the process of coding the spatial-related content. A code was given to the spatial-related content by either extracting the original words from the text (i.e., in-vivo coding) or creating new words based on the content context (descriptive content). In total, the coding process yielded 44 unique codes. Table 2 shows examples of in-vivo and descriptive coding on the identified spatial-related content of Table 1.

**Table 2:** Examples of in-vivo and descriptive coding.

Spatial-related content	Codes	Coding method
• ^a1^Orientation in the local environment and the^a2^structure of simple^a3^maps. • Concepts that describe^b1^location,^b2^distance and^b3^direction. (Skolverket, 2024, p.182).	^a1^ Orientation	In-vivo coding
	^a2^ Structure	In-vivo coding
	^a3^ Maps	In-vivo coding
	^b1^ Location	In-vivo coding
	^b2^ Distance	In-vivo coding
	^b3^ Direction	In-vivo coding
• ^c1^ The structure, cycle and indestructibility of matter visualised using particle^c2^models. • ^d^ Elements, molecular and ionic compounds and how substances are transformed through chemical reactions. • ^e^ Atoms, electrons and nuclear particles. (Skolverket, 2024, p.169).	^c1^ Structure	In-vivo coding
	^c2^ Models	In-vivo coding
	^d^ Object structure	Descriptive coding
	^e^ Object structure	Descriptive coding

Third, we adapted Lin and colleagues’ [18] two-dimensional framework to explore possible patterns among the 44 codes. We included another component, inductively described as “spatial citizenship knowledge”, representing the spatial information that pupils use to deliver location-specific opinions to participate in society [44]. The new component was deemed essential as it permeates many subjects beyond those Lin and colleagues [18] examined (i.e., Craft and Technology). For example, the terms such as “structure” and “movement” were considered to inherit the spatial attributes, and pupils were expected to transfer these attributes to a historical context and deliver opinions regarding “social structure” and “population movement”. In this study, we also re-termed what Lin and colleagues [18] termed “knowledge types” to “epistemic dimension” to encompass a broader range of spatial knowledge. Moreover, this new term may better correspond phonetically with the “visual dimension”.

Fourth, we synthesised the two dimensions, namely, the visual and epistemic dimensions. The visual dimension includes the three components that are suggested by Lin and colleagues' [18] framework, which are “graphical”, “pictorial”, and “manufactured”, and the epistemic dimension contains the components “conceptual” and “procedural” from Lin and colleagues’ [18] study, and the newly introduced “spatial citizenship knowledge”. Table 3 shows the two dimensions and the definitions of their components.

Fifth, a three-by-three matrix framework was built based on the two dimensions reported above. For the matrix (Table 4), the columns represent the visual dimension, and the rows represent the epistemic dimension. Correspondingly, the cells represent the spatial-related content that demonstrates the combinations of the visual components and epistemic components. An example of the spatial-related content corresponding to each cell of the matrix is available in Appendix A. The examples provide concrete depictions of how the content related to “graphical-conceptual”, for example, is represented in the curriculum.

**Table 3:** Definitions of the components of the two dimensions.

Component	Definition
Visual dimension	
Graphical	“… schematic features such as “symbol” and “graph”. They are in a two-dimensional form with the essential attributes that show the details of the structure, framework, and construction of an entity” ([18], p5).
Pictorial	“… contextual features such as “picture” and “material”. They are in a two-dimensional form with not only schematic features but also more aesthetic details such as colours and texture” ([18], p5).
Manufactured	“… a three-dimensional entity which combines both graphical and pictorial components such as “handicraft” and “artefacts”” ([18], p5).
**Epistemic dimension**	
Conceptual	“Knowledge that pupils acquire about the concepts, principles, as well as cultural and historical facts of an entity” ([18], p5).
Procedural	“Knowledge that pupils are applying while carrying out hands-on activities to reach a solution of a problem” ([18], p5).
Spatial citizenship	Spatial information that pupils used to deliver location-specific opinions to participate in society.

Finally, we situated all subjects in the three-by-three framework with the frequency of codes.

### 3.3 Ethical considerations

We collected the data from a publicly accessible curriculum document. People, human tissue, or sensitive personal data covered by the Swedish Ethics Act were not involved or addressed by this study.

## 4 Results

The findings will be reported according to various subject categories including languages learning, social studies, STEAM (i.e., STEM and Art), and other subjects. First, we present the total frequency of the codes as well as the frequency of unique codes captured in each cell. By presenting both the frequency of total codes and unique codes it allows both the proportions and the varieties of the spatial-related content in each cell to be understood. Second, we plotted the differences between the three grade stages (i.e., years 1–3, years 4–6, and years 7–9) regarding each subject category.

### 4.1 Languages learning

The potential to identify and interpret spatial ability in languages learning (subjects include English, Swedish, Swedish as a Second Language, Modern Languages, Mother Tongue, Sami, and Sign Language for the Hearing) is represented in all of the cells (Table 5).

**Table 4:** The three-by-three framework.

		Visual dimension		
		Graphical	Pictorial	Manufactured
**Epistemic dimension**	**Conceptual**	Graphical-conceptual	Pictorial-conceptual	Manufactured-conceptual
	**Procedural**	Graphical- procedural	Pictorial-procedural	Manufactured- procedural
	**Spatial citizenship**	Graphical-spatial citizenship	Pictorial-spatial citizenship	Manufactured-spatial citizenship

Despite the comparatively large total frequency of the codes, the varieties of how languages learning could be interpreted into pupils' spatial ability development is limited when considering unique codes. Most language subjects demonstrate spatial-related content by requiring students to identify language “structure” or alphabetical “order” (graphical-conceptual). Some language subjects could potentially develop pupils’ spatial ability by requiring them to compare language variants used by different countries in the world (graphical-conceptual).

It is worth mentioning that the comparatively high frequency of Modern Languages and Mother Tongues should be attributed to combining all elective languages they encompass. Specifically, the Modern Languages subject includes all the elective languages for pupils to choose such as Chinese. In addition, the Mother Tongues include Finnish, Meänkieli, Romani Chib, and Yiddish. The spatial-related content of these languages does not differ much from each other, and the knowledge requirements are highly similar. To give an example, while the subject of Finnish for years 1–3 requires pupils to understand “The Finnish alphabet and the relationship between sounds and letters.” ([16], p.98), Yiddish requires pupils to acquire “The printed alphabet and the relationship between sounds and letters” ([16], p.141). Therefore, the aggregate frequency of Modern Languages and Mother Tongues shown in the cells may not necessarily reflect a substantial portion of the subjects.

It is also worth noticing that Sign Language for the Hearing presents a large degree of potential opportunities to develop pupils’ spatial ability. This is because sign language inherently possesses a spatial nature, leading pupils to engage in visual-spatial integration during practice. As stated in the document, for instance, pupils who learn Sign Language for the Hearing should attain “Basic principles of the gestural-visual structure of sign language” ([16], p.204).

**Fig. 2:**
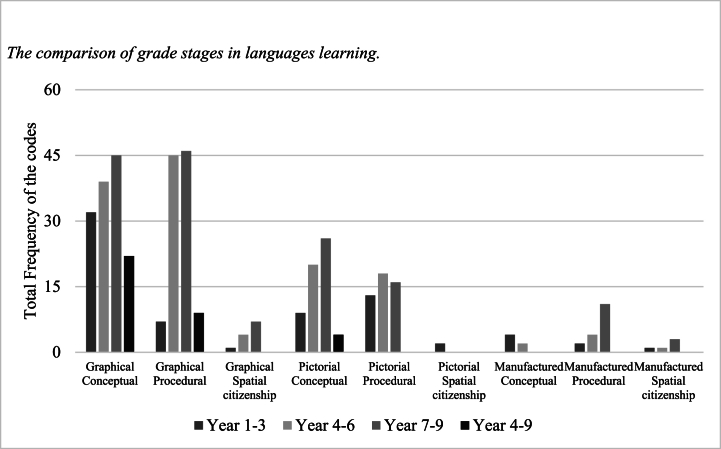
The comparison of grade stages in languages learning.

In terms of grade stages, most of the language subjects are delivered across three stages, namely, years 1–3, 4–6, and 7–9. Exceptionally, the Modern Language subjects and Sign Language for the Hearing divide the content for year 4–6, 7–9, and 4–9. These are the only subjects to present an additional section of content for years “4–9”, which could be to reflect a view that learning languages involves consistent immersion in certain activity such as conversation and reading. No explanation for the different structure is provided in the document, so it should be noted for the presented results that this overlap exists for these subjects. Fig. 2 shows that the spatial-related content of Languages Learning occurs across all the grade stages, but the proportions for years 4–6, 4–9, and 7–9 are higher than years 1–3. All grade stages most likely develop spatial ability through the graphical and pictorial components with similar amounts of conceptual and procedural knowledge.

### 4.2 Social studies

Social Studies in the Swedish national curriculum includes Geography, Civics, History, and Religion. The potential to consider Geography in terms of pupils' spatial ability development occurs across most of the components. In comparison to other social studies subjects, Geography has the potential to develop pupils’ spatial ability in a more diversified way as its number of unique codes is shown. Other social studies subjects such as History, Civics, and Religion mainly capture the epistemic dimension of spatial citizenship knowledge (Table 6). These subjects describe several spatial-related terms in the social context such as “structure”, “movement”, and “relationship”, requiring students to understand social issues and cultural events by comparing different countries in the world. For example, in Civics, pupils of years 7–9 compare how human rights are practiced in different countries, through which teachers could potentially design a pedagogy to involve a mental process of regarding different countries as objects and identifying a reference between the source and target objects (graphical-spatial citizenship).

**Table 5:** The three-by-three framework for languages learning.

		Visual dimension		
		Graphical	Pictorial	Manufactured
**Epistemic dimension**	**Conceptual**	English (5,1)	English (1, 1)	Sami (6, 3)
		Modern Language (22, 1)	Modern Language (2, 1)	
		Mother Tongues (70, 2)	Mother Tongues (8, 2)	
		Sami (6, 1)	Sign Language for the Hearing (26, 2)	
		Swedish (10, 1)	Swedish (4, 1)	
		Swedish as a Second Language (14, 1)	Swedish as a Second Language (5, 1)	
		Sign Language for the Hearing (5, 3)	Sami (15, 1)	
	**Procedural**	Sami (31, 2) Sign Language for the Hearing (8, 1) Swedish (14, 2) Swedish as a Second Language (21, 2) Modern Language (12, 2) Mother Tongues (20, 2)	English (2, 2) Sami (8, 2) Sign Language for the Hearing (8, 1) Swedish (7, 1) Swedish as a Second Language (18, 1)	Sami (15, 2) Swedish (2, 1)
	**Spatial citizenship**	Sami (9, 3)	Swedish (1, 1)	Swedish (5, 2)
		Swedish (3, 2)	Swedish as a Second Language (1, 1)	

Note.

(1) In the brackets, the first number represents the total frequency of the codes, and the second number represents the frequency of the unique codes.

**Table 6:** The three-by-three framework for social study subjects.

		Visual dimension		
		Graphical	Pictorial	Manufactured
**Epistemic dimension**	**Conceptual**	Geography (29, 7)		
		Civic (3, 3)		
		History (2, 1)		
	**Procedural**	Geography (15, 3)	Geography (1, 1)	
	**Spatial citizenship**	Civic (29, 2) History (8, 1) Geography (6, 3) Religion (2, 1)	Civic (7, 1) Geography (1, 1)	History (6, 1) Religion (2, 1)

Note.

(1) In the brackets, the first number represents the total frequency of the codes, and the second number represents the frequency of the unique codes.

**Fig. 3:**
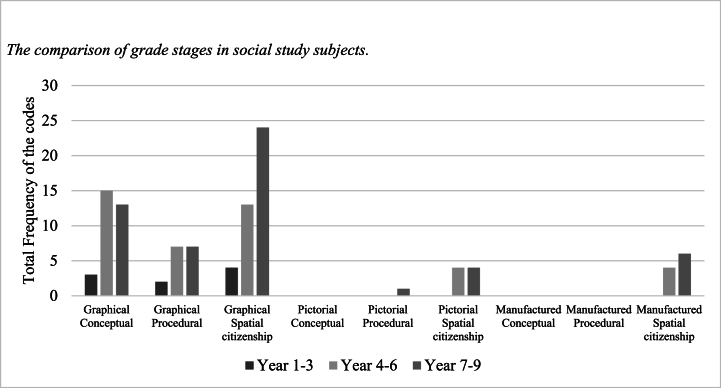
The comparison of grade stages in social study subjects.

In terms of grade stages (Fig. 3), the spatial-related content of social study subjects is more developed in years 4–6 and 7–9. In addition, all grade stages cluster in the graphical components of the visual dimension. A small amount of the spatial-related content is developed through pictorial- and manufactured-spatial citizenship by years 4–6 and 7–9.

### 4.3 STEM + Arts (STEAM)

Mathematics, Chemistry, Biology, Physics, Technology, Craft, and Art are grouped into the STEAM category. Spatial-related content in STEAM subjects mostly concerns the graphical and pictorial components of the visual dimension, in conjunction with conceptual and procedural knowledge (Table 7) on the epistemic dimension. Mathematics is found to have the greatest amount of spatial-related content due to its inclusion of geometrical knowledge that is inherently spatial. In addition, according to previous studies (e.g. Ref. [48]), performing Boolean operations in Mathematics such as difference, union, and intersection holds a great opportunity to develop pupils’ spatial ability.

**Table 7:** The three-by-three framework for STEAM learning.

		Visual dimension		
		Graphical	Pictorial	Manufactured
**Epistemic dimension**	**Conceptual**	Mathematics (72, 5) Chemistry (28, 1) Craft (3, 3) Technology (9, 1) Biology (9, 2) Art (8, 8) Physic (8, 1)	Art (15, 9) Craft (12, 2) Mathematics (1, 1) Technology (1, 1)	Art (14, 8) Craft (27, 3) Mathematics (1, 1) Technology (7, 3)
	**Procedural**	Mathematics (41, 5) Craft (3, 4) Technology (19, 3) Art (19, 8) Chemistry (6, 2) Biology (5, 2) Physic (4, 1)	Art (39, 11) Technology (1, 1) Craft (4, 2) Chemistry (9, 1) Physic (9, 1) Biology (9, 1)	Craft (18, 3) Art (1, 1) Technology (13, 3)
	**Spatial citizenship**	Chemistry (1, 1) Art (1, 1)	Art (2, 2)	Art (1, 1)

**Note:** In the brackets, the first number represents the total frequency of the codes, and the second number represents the frequency of the unique codes.

Art also yields a comparatively large number of spatial-related content. The required repertoire of Art exposes pupils to all visual components. Pupils do not only need to understand and evaluate artistic artefacts but also to create such artefacts by hand. Moreover, the number of unique codes generated in Art is larger than any other subject in the curriculum, showing its great inclusiveness for spatial ability development.

Other STEAM subjects such as Physics, Biology, Chemistry, Technology, and Craft could potentially develop pupils' spatial ability by requiring an understanding of object structure (graphical-conceptual), such as the particle model required in Chemistry, organ structure in Biology, and computer structure in Technology. In addition, the hands-on activities offered by STEAM subjects allow pupils to develop spatial ability as related to fine motor skills. For example, acquiring handicraft techniques such as woodwork could foster pupils’ visualization (manufactured-procedural).

**Fig. 4:**
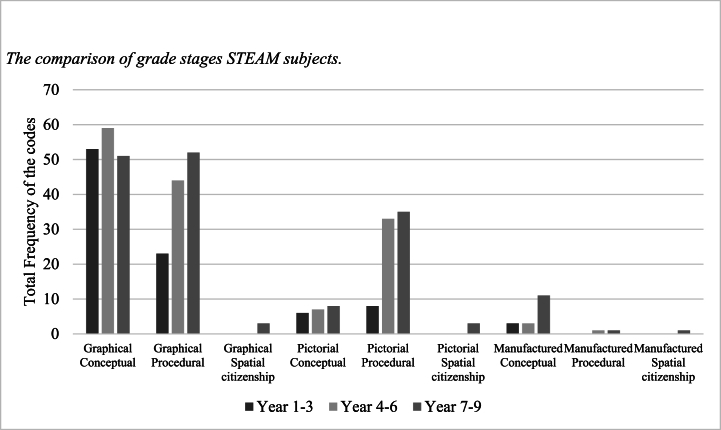
The comparison of grade stages STEAM subjects.

Graphical- and pictorial-conceptual components occupy a similar amount of spatial-related content among the three grade stages (Fig. 4). For other components, spatial-related content is more developed in years 4–6 and 7–9 than in years 1–3. In general, all grade stages emphasize graphical components over pictorial and manufactured.

### 4.4 Other subjects

Other subjects include Music, Home and Consumer Studies, and Physical Education (PE) (Table 8). Music and PE could potentially develop pupils’ spatial ability to a great extent, and they demonstrate a similar number of unique codes. In Music, pupils learn to recognize musical symbols and notes and play musical instruments. These symbols and notes are often shown in structure and patterns (graphical-conceptual). Furthermore, pupils need to be familiar with the structure of the instruments (graphical-conceptual). In PE, pupils are supposed to practice complex physical movement in games and sports, during which dynamic, fine motor skills and spatial orientation are involved (graphical-procedural). Additionally, outdoor life and activities are highlighted in PE class, where pupils engage in orienteering and navigate in different environments with maps (graphical-procedural).

**Table 8:** The three-by-three framework for other subjects.

		Visual dimension		
		Graphical	Pictorial	Manufactured
**Epistemic dimension**	**Conceptual**	PE (3, 3)	Music (10, 5)	
		Music (6, 4)		
	**Procedural**	PE (33, 4)	Music (5, 2)	Music (6, 1)
		Music (23, 2)		
	**Spatial citizenship**			

Note.

(1) In the brackets, the first number represents the total frequency of the codes, and the second number represents the frequency of the unique codes.

We did not identify any apparent spatial ability development in the subject of Home and Consumer Studies. However, it does not necessarily exclude the potential to develop pupils' spatial ability within the subject. Home and Consumer studies aim to foster pupils' interest and knowledge in making choices pertaining to health, finances, and the environment. The subject involves knowledge and activities regarding food and cooking, as well as personal finances and consumption. There is a likelihood that pupils may use their spatial ability during the process of cooking by following the instructions and recipes, sorting out ingredients, manipulating utensils and weighing scales. These potential activities may develop pupils’ visual-spatial integration. In addition, mental Boolean operations may be utilized as pupils make financial decisions. While we foresee the possibility of these activities, nevertheless, we refrain from over-interpreting the curriculum content due to the absence of explicit spatial-related terms.

**Fig. 5:**
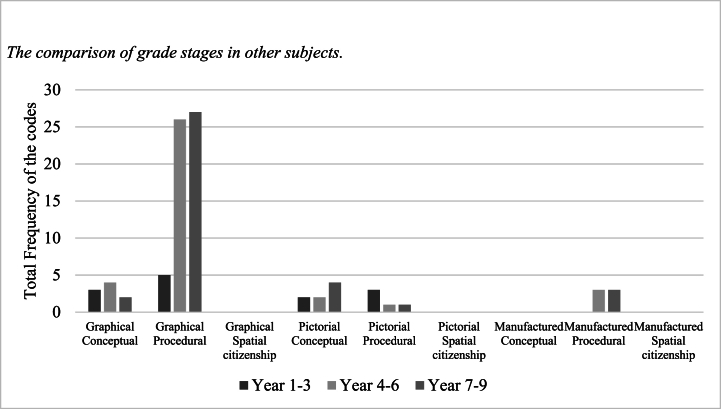
The comparison of grade stages in other subjects.

Music and PE largely cluster in graphic-procedural components among all grade stages (Fig. 5). Spatial-related content appears more in years 4–6 and 7–9 than in years 1–3 across all available components except pictorial-procedural.

## 5 Discussion

Spatial-related content is identified in all of the subject syllabi to various extents except for the subject of Home and Consumer Studies. The developed three-by-three framework further enables it to be seen that potential spatial ability development in the Swedish national curriculum mostly relates to the graphical component of the visual dimension, and the conceptual and procedural components of the epistemic dimension. In addition, most of the spatial-related content is found to be developed in years 4–6 and 7–9 rather than years 1–3. The findings answered our research question– how can spatial ability be potentially identified and interpreted in the Swedish national curriculum?

### 5.1 Practical implications

#### 5.1.1 Applying the three-by-three framework

The three-by-three framework offers a practical tool for educators and policymakers to support the current practice in teaching concerning spatial ability development. With the framework, teachers can identify the visual components of the curriculum and understand the knowledge type that pupils are supposed to acquire. Teachers can then alternate between components and adopt spatialized pedagogies to potentially optimize the effect of spatial ability development.

When teachers apply the visual dimension of the three-by-three framework, it is recommended that they transform components to facilitate pupils’ holistic spatial ability development. For example, when Art teachers present architecture (a manufactured component) from different cultures, they could guide pupils to compare the materials (pictorial component) as well as the shapes, sizes, and lines (graphical components) of the architecture. Similarly, in Mathematics lessons on two-dimensional shapes such as squares and triangles (graphical components), teachers could help pupils identify these shapes in three-dimensional objects familiar in daily life, such as desks and stools (pictorial and manufactured components) found in the classroom. This approach aligns with spatial factors such as spatial visualization (e.g. Ref. [22]), spatial orientation (e.g. Ref. [23]), and spatial relations (e.g. Ref. [24]), which are often engaged when pupils work with multiple visual components. By engaging with various visual components of the three-by-three framework, pupils are likely to visualize and perceive the objects from various perspectives and relate one perspective to another. Consequently, subjects that incorporate more diverse components might have more impact on pupils' holistic spatial ability development. Our findings indicate that certain subjects exhibit a greater variety of components than others. For example, although Language Learning includes numerous components related to language structure (graphical component), Social Studies and STEAM subjects offer more diverse opportunities for spatial ability development. In Geography, for example, pupils can enhance their spatial ability through activities such as learning about the sizes of geographical objects (graphical and manufactured components), the relationships between them (graphical and manufactured components), and the scales of geographical maps (graphical and pictorial components).

In the epistemic dimension of the three-by-three framework, it is suggested that teachers transform conceptual and spatial citizenship components into procedural components. For example, when pupils learn about demographic patterns (a conceptual component) in Geography, teachers could have them draw mind maps (a procedural component) to describe the population distribution and trends. This is because spatial factors involving dynamic and fine motor skills have the most direct effect on developing spatial ability [26]. Our study shows that procedural knowledge is emphasized across most subjects in the Swedish national curriculum, presenting numerous opportunities for teachers to enhance pupils' spatial ability. These opportunities often manifest through visual-spatial integration, which is frequently required in STEAM subjects such as Art, Craft, Mathematics, and Technology. Physical Education also contributes to visual-spatial integration, as students engage in physical movements through games and sports in various environments [29]. Additionally, in Language Learning, students can develop visual-spatial integration through writing tasks, which require them to visually inspect and retain alphabetical and grammatical structures in mind and then reproduce them using motor movements—writing.

The three-by-three framework provides teachers across all subjects with a structured approach to identify opportunities to enhance pupils' spatial ability. Nevertheless, the application of the framework appears rather implicit within certain Social Studies subjects, such as Civics, History, and Religion. These subjects primarily encompass spatial citizenship knowledge, offering much freedom for teachers to determine the methods and extent to which they develop pupils' spatial ability. For example, when discussing social phenomena in various countries, teachers can leverage these opportunities to engage students in navigating the geographical locations of the countries, describing the spatial relationships between the countries, or drawing mind maps that involve both subject-specific and spatial knowledge. However, without adequate awareness of spatial ability development, teachers might overlook these opportunities. Policymakers can play a crucial role here by incorporating more explicit language pertaining to visual components within the framework. Such explicit language could enhance teachers' awareness of spatial ability development, thereby maximizing the framework's effectiveness. Therefore, policymakers should consider including clearer guidelines related to spatial ability development across the framework's components.

#### 5.1.2 Developing spatial ability for early grades

Our findings indicate that spatial-related content is more prominent in the later stages of the Swedish national compulsory curriculum than in the early stages, which may not align with the best practices suggested by previous research. It is desirable for educators and policymakers to increase or make more explicit the spatial-related content also for pupils in years 1–3.

Previous studies suggest that early spatial ability performance can predict later academic achievement, particularly in mathematics (e.g. Refs. [49,50]). For example, the ability to distinguish mirrored images from non-mirrored images in infancy is a promising precursor of mathematical performance at four years of age [51]. A longitudinal study with Finnish children found that spatial ability measured in kindergarten (around six years old) predicted arithmetic performance from first grade (around eight years old) through third grade (around ten years old) [50]. Similarly, Verdine and colleagues [49] found that the spatial ability of preschoolers at three years old predicted significant variation in their mathematics problem-solving at five years old. Furthermore, Gilligan and colleagues [52] found that spatial ability at five years old predicted mathematics performance at seven years old.

Importantly, early spatial training is a gatekeeper for gender diversity in the STEM field (e.g. Ref. [7]). Gender differences in spatial ability, such as mental rotation, tend to widen with age [53]. Specifically, male students have been found to outperform females in some spatial tasks as they grow older [53]. Since spatial ability is one of the strongest predictors of STEM educational achievement, these growing gender differences might further discourage female students from pursuing STEM subjects in the future [7]. Therefore, it is essential to include more explicit guidelines for developing pupils' spatial ability in the curriculum for years 1–3.

### 5.2 Limitations

The three-by-three framework was built through a bottom-up interpretation of the curriculum document, presenting various inductively derived spatial dimensions and components. This approach contrasts with most spatial ability research, which typically focuses on established spatial factors such as spatial visualization and spatial orientation. Thus, this approach may seem unconventional to previous spatial ability researchers. However, the intention behind this approach was to understand the national curriculum from the perspectives of educators and policymakers rather than starting from a researcher's viewpoint. By examining the document through a bottom-up approach, the three-by-three framework aims to increase educators' and policymakers' awareness of the potential for developing spatial abilities within the existing curriculum that they are familiar with. This helps them recognize that spatial ability is already embedded in their current practices, thereby avoiding additional workload.

In addition, we cannot draw any conclusions about the curriculum document's effects on pupils' actual performance or how teachers implement these practices. Echoing Örbring's [17] study, since spatial ability is rather implicit in the curriculum, its development among pupils largely depends on teachers' interpretations. To support this inquiry, we created the three-by-three framework to provide educators with an exercisable tool for interpreting the document.

## 6 Conclusion

It is an enduring and complex journey for stakeholders to enhance the efficiency of educational documents on teachers’ enacted practice. This is speaking for not only spatial ability development but also other disciplinary domains in education. National curriculum document is issued following certain educational traditions and is largely shaped by national perspectives. Future research could utilize the three-by-three framework to (a) examine the prevalence of spatial ability in the national curriculum of various countries, enabling the construction of a more generic framework applicable to broader educational contexts; and (b) explore the connections between previously studied spatial factors and the components of the three-by-three framework, thereby generating more empirical evidence to support the framework.

## Data availability statement

Data associated with this study are publicly accessible at Skolverket. (2024). *Curriculum for Compulsory School, Preschool Class and School-Age Educare|: Lgr 22. Stockholm*. https://www.skolverket.se/publikationer?id=12435.

## CRediT authorship contribution statement

**Ting Jun Lin:** Writing – original draft, Visualization, Software, Methodology, Investigation, Formal analysis, Data curation, Conceptualization. **Jeffrey Buckley:** Writing – review & editing, Validation, Supervision, Methodology, Investigation, Data curation. **Lena Gumaelius:** Writing – review & editing, Validation, Supervision, Project administration, Investigation, Funding acquisition. **Ernest Ampadu:** Writing – review & editing, Validation, Supervision, Project administration, Investigation.

## Declaration of competing interest

The authors declare the following financial interests/personal relationships which may be considered as potential competing interests:Ting Jun Lin reports financial support was provided by 10.13039/501100000780European Union. If there are other authors, they declare that they have no known competing financial interests or personal relationships that could have appeared to influence the work reported in this paper.

## Figures and Tables

**Table:** 

Matrix	Example (Subject and the curriculum content)
Graphical-conceptual	Mathematics
	Basic geometric **two-dimensional objects** and the objects **sphere, cone, cylinder and cuboid** ([16], p.58)
Graphical-procedural	Chemistry
	The **structure** of matter visualised using simple particle **models** ([16], p.169).
Graphical-spatial citizenship	Geography
	Causes and consequences of demographic **patterns** and changes:
	population distribution, population trends, migration patterns and
	urbanisation ([16], p.223)
Pictorial-conceptual	Crafts
	Metal, **textiles** and wood. Properties and uses of **materials** ([16], p.38).
Pictorial-procedural	Science (for years 1–3)
	Performing and documenting the investigations with words, **images** and digital tools. ([16], p.162).
Pictorial-spatial citizenship	Languages learning—Swedish
	Fiction for children from different times and **different parts of the world**. Songs, oral narration, **picture books**, chapter books, lyrical poetry, drama, fairy tales and myths. ([16], p.244).
Manufactured-conceptual	Languages learning—Sami
	Sami cultural heritage in various forms, such as games, music, **handicraft** and other forms of aesthetic expression ([16], p.190).
Manufactured-procedural	Technology
	The pupils' own **constructions** using mechanisms, electrical connections and strong and stable structures ([16], p. 269).
Manufactured-spatial citizenship	Art
	**Fine art pieces**, documentary art and **architecture** from different times and cultures. ([16], p.33)

**Note:** The spatial attributes of the content are indicated by bold words.
